# Theoretical perspective on synthetic man‐made life: Learning from the origin of life

**DOI:** 10.1002/qub2.22

**Published:** 2023-11-27

**Authors:** Lu Peng, Zecheng Zhang, Xianyi Wang, Weiyi Qiu, Liqian Zhou, Hui Xiao, Chunxiuzi Liu, Shaohua Tang, Zhiwei Qin, Jiakun Jiang, Zengru Di, Yu Liu

**Affiliations:** ^1^ International Academic Center of Complex Systems Beijing Normal University Zhuhai China; ^2^ Swarma Research Beijing China; ^3^ Department of Philosophy Shanghai Jiao Tong University Shanghai China; ^4^ Center for Biological Science and Technology Advanced Institute of Natural Sciences, Beijing Normal University Zhuhai China; ^5^ Center for Statistics and Data Science Beijing Normal University Zhuhai China

**Keywords:** artificial cell, autocatalytic, ladderpath, origin of life, protocell

## Abstract

Creating a man‐made life in the laboratory is one of life science’s most intriguing yet challenging problems. Advances in synthetic biology and related theories, particularly those related to the origin of life, have laid the groundwork for further exploration and understanding in this field of artificial life or man‐made life. But there remains a wealth of quantitative mathematical models and tools that have yet to be applied to this area. In this paper, we review the two main approaches often employed in the field of man‐made life: the top‐down approach that reduces the complexity of extant and existing living systems and the bottom‐up approach that integrates well‐defined components, by introducing the theoretical basis, recent advances, and their limitations. We then argue for another possible approach, namely “bottom‐up from the origin of life”: Starting with the establishment of autocatalytic chemical reaction networks that employ physical boundaries as the initial compartments, then designing directed evolutionary systems, with the expectation that independent compartments will eventually emerge so that the system becomes free‐living. This approach is actually analogous to the process of how life originated. With this paper, we aim to stimulate the interest of synthetic biologists and experimentalists to consider a more theoretical perspective, and to promote the communication between the origin of life community and the synthetic man‐made life community.

*What I cannot create, I do not understand.*

—On Richard Feynman’s blackboard

## 1 INTRODUCTION

How to create life in a laboratory is one of the most intriguing problems in life science. The “modern” beginning of the origin of life research started with Miller‐Urey in the 1940s [1], clearly showing that inorganic can be transformed into organics, and there are no fundamental barriers between life and non‐life. Since then, people realized that creating a simple “alien” life—namely, a simple life form that never existed on Earth—is at least possible. This type of alien life may be referred to as man‐made life, synthetic life, or wet artificial life (the term “artificial life” often particularly refers to the studies in silico about the fundamental processes of living systems), if emphasizing its “man‐made” property; or referred to as artificial cell, synthetic cell, minimal cell, or protocell, if emphasizing its “cell” property. In this paper, we just use the loose term “man‐made life” without distinguishing them in particular. On the one hand, some people try to create man‐made life according to the extant forms of life on Earth, trying to reduce the complexity of living systems (from the extant complex life) to a minimal level so that the most basic and essential requirements of life can be revealed. This is thus often referred to as the top‐down approach. On the other hand, others try to find conditions for defining life and create systems that satisfy the conditions, namely, integrating well‐defined components. As this approach tries to locate the most basic form of life, it is thus often referred to as the bottom‐top approach.

Thanks to the rapid development of synthetic biology, the top‐down approach has made substantial contributions to man‐made life. In fact, people have succeeded in “creating” life in laboratories, for example, [2, 3]. Nevertheless, what the approach has achieved is not creating life from non‐life, but from life. It uses materials produced by life to assemble a living cell. Even though it is an outstanding achievement and contributes to our understanding of the essential properties of life, this approach presupposes the very existence of life. Moreover, the life forms presupposed by the approach are products of hundreds of millions of years of evolution since the origin of life. Erasing the evolutionary history of life may describe a misleading picture of the nature of life. Some of the functions owned by the current life forms may not be essential to the simplest and most primary life.

The bottom‐up approach tries to find the blueprint of life and conditions for defining life. Scholars have long found that it is almost empirically impossible to find properties that are individually necessary and jointly sufficient for defining life [4, 5]. What we need may not be a definition but a theory of the essential properties of life [6]. Nevertheless, the properties mostly observed from life on Earth constitute a starting point for scientists. Generally speaking, metabolism/self‐replication/self‐maintaining (or self‐sustaining), information, and compartment/individuality are the most cited properties which people think constitute the nature of life. Accordingly, we classify various proposed theories of the properties of life in Table 1 (not complete but a representative table):

1. Erwin Schrödinger’s concept of life from a thermodynamic perspective, as outlined in his famous book “*What is Life*?”, involves the idea that life feeds on a constant inflow of negative entropy, allowing it to have metabolic processes and self‐replicate [7]. In thermodynamics, the degree of disorder or randomness in a system is measured by entropy. Thus, in this context, “feeding on negative entropy” can be understood as a metaphor for acquiring information or organization from the external environment.
2. In the 1970s, Tibor Gánti proposed that living systems must possess three fundamental subsystems: a membrane‐like structure that serves as a boundary to separate the system from its environment, a metabolic subsystem for the replenishment and renewal of nutrients, and a subsystem for the inheritance of traits [8]. The last two subsystems align with Schrödinger’s concept, while he emphasized the crucial role of the boundary or compartment: The second law of thermodynamics dictates that the entropy of a closed system will naturally increase over time, but the organization of living systems necessitates the presence of a boundary to separate itself with the environment [9]. This concept is referred to as “Chemoton”.
3. *Autopoiesis* was proposed by Humberto Maturana and Francisco Varela [10]. Since they were not satisfied with molecular biology, the mainstream approach for studying living systems, which argued that genetic information is essential to life, they developed the theory of *autopoiesis* according to the organization of cells which are basic units of life. Individuality and interaction with the environment are their primary concern, not information.
4. NASA (National Aeronautics and Space Administration) has a working definition of life: a self‐sustaining chemical system capable of Darwinian evolution [11, 12, 13]. This definition was often credited to Gerald Joyce, who summarized the discussion of a committee set up by NASA in 1994 that aims to give a definition of life. This definition is generally accepted as a useful, albeit somewhat general definition, but the involved terms “self‐sustaining” and “Darwinian evolution” themselves have complex definitions and may require further clarification. This definition emphasizes Darwinian evolution in particular, so we also only emphasize that in Table 1. Although we understand that many researchers agree that Darwinian evolution may imply self‐replication and information/genetics (and also mutation/variation), we do not mark down these implications.
5. According to Pier Luigi Luisi, life should be understood as a system that is defined by its semi‐permeable chamber structure, which is able to sustain itself through its own internal processes, involving selectively exchanging nutrients and energy with its environment. This perspective emphasizes the importance of compartments, energy, and metabolism (in particular, the property of self‐sustaining) [14, 15].
6. A 250‐page document “NASA Astrobiology Strategy 2015” released by NASA pointed out that the advent of polymers is critical for the emergence of life, and the polymers must be able to replicate, store genetic information, and be capable of Darwinian evolution [16]. This emphasizes the importance of polymers, as a particular type of macromolecule.
7. A “*Nature*” special issue “Bottom‐up Biology” summarized that the essential factors for creating life from scratch are compartmentalization, energy, and information molecules [17]. In other words, it is suggested that these three factors are necessary for the emergence of life.

**TABLE 1:** Various proposed theories on the properties of life.

References				Metabolism				Information/Genetics	Compartment/Individuality	Darwinian evolution	Other
					Emphasis on:						
					Self‐replication	Self‐sustaining	Energy				
1	In “*What is life*?”	Schrödinger, 1944	[7]	√	√			√			
2	Chemoton	Gánti, 1970s	[8]	√				√	√		
3	Autopoiesis	Maturana & Varela, 1972	[10]	√		√			√		
4	NASA’s working definition of life	Joyce et al., 1994	[11, 12, 13]			√				√	√
5		Luisi, 1998	[14, 15]			√	√		√		
6	In “NASA astrobiology strategy 2015”	NASA, 2015	[16]		√			√		√	√
7	In a special issue of “*Nature*”	Powell et al., 2018	[17]				√	√	√		

*Note*: “Self‐replication” refers to the ability of a living system to make copies of itself. “Self‐sustaining” refers to the ability of a living system to regenerate all of its components, to maintain its existence and functionality over time (in fact, if a system is able to self‐replicate, it must be self‐sustaining under other conditions, and vice versa, see details in Section 3.1). “Energy” refers to the ability to acquire and use energy to fuel metabolic processes. The three all belong to “Metabolism”. “Information/Genetics” refers to the presence of genetic material that encodes information and can be passed on to offspring. “Compartment/Individuality” refers to the presence of a physical boundary that separates the system from its environment and allows for individual identity. “Darwinian evolution” refers to the ability to undergo heritable changes over time, resulting in adaptations that increase the chances of survival and reproduction. Finally, in the 4th row, “Other” property refers to “chemical system”; In the 6th row, “Other” property refers to “polymers” (see explanations in main texts).

**FIGURE 1:**
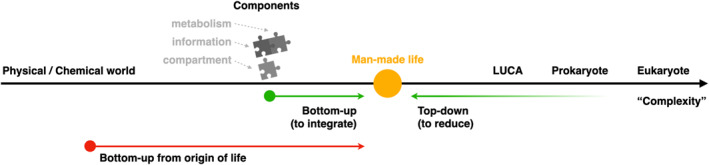
The sketchy research map of “man‐made life”.

Although the aforementioned properties of life are not obviously consistent, they are not mutually exclusive but looking at the question from slightly different angles. In principle, if we are able to integrate these properties or factors into a system, the goal of creating man‐made life is achieved. The top‐down method succeeds by borrowing the factors from extant life and recombining them, while the bottom‐up method approaches the goal by synthesizing and creating these factors de novo in the laboratory and trying to integrate them (referring to Figure 1). In Section 2.1, we will talk about the top‐down approach, which has actually succeeded in a way. On the other hand, although the bottom‐up method has not achieved the ultimate goal of creating a man‐made life in a real sense, it has made substantial progress for each aspect of the factors shown in Table 1. We will discuss them, respectively, in detail in Section 2.2.

In fact, it could be possible to push the bottom‐up approach a bit further, by realizing and appreciating the rich results collected in the past decades in a strongly related community, the “origin of life” community (there we focus on the underlying mechanisms that make the emergence of life possible, but not the historical problems such as where and when exactly life on Earth started). If those concepts, theories, and tools could be exploited and used in the task of man‐made life, this approach could still be called the “bottom‐up” approach, where we have just pushed the “bottom” further to the non‐living (namely physical and chemical) world, referring to the red arrow “Bottom‐up from the origin of life” in Figure 1. Indeed, there were not many communications between the two communities, probably because there seemed to be a wide gap between the theories/concepts proposed in the origin of life research and the dirty chemical/biological world the synthetic scientists have to face when trying to create man‐made life. But thanks to the rapid development of synthetic biology and related theoretical works, the gap is shrinking, and it is time. In Section 3, we will discuss the profound theories and hypotheses in the field of the origin of life, namely the autocatalytic sets theory and related ones, but also the problems we have to face if this concept is borrowed to man‐made life (Section 3.1). After that, we will try to give our attempted answers to these problems (Sections 3.2 and 3.3). Finally, in Section 4, we draw the discussion and conclusion.

## 2 THE ENDEAVOR: TOP‐DOWN AND BOTTOM‐UP APPROACH

### 2.1 Top‐down: Reducing complexity

The top‐down approach for man‐made life involves reducing the complexity of extant and existing living systems, in order to identify the minimal requirements for basic life functions. This approach has been employed by a number of researchers, with typical examples shown below.

- Hack living cells: From JCVI‐syn1.0 to JCVI‐syn3A

In 2010, Venter’s team synthesized the genome of the bacterium *Mycoplasma mycoides* from scratch and inserted it back into an *M*. *mycoides* cell that had previously had its genome removed. After rounds of divisions, normal cells and cells containing only the artificial genome emerged. The normal cells were then killed with antibiotics, and the “artificial cells” were then obtained, named JCVI‐syn1.0 or Synthia [18]. But in order to ultimately design a minimal genome containing only the essential genetic information required for life, Venter’s team moved on, with the specific “design‐build‐test” cycles, and eventually announced the synthesis of JCVI‐syn3.0 in 2016. It has a genome containing 531 kilobase pairs (473 genes), smaller than any cell found in nature that is capable of independent self‐replication [2, 19]. However, JCVI‐syn3.0 has many morphological variations, such as filamentous and vesicular forms, which were unexpected and seemed “abnormal”. To solve this problem, in 2021, Pelletier et al. designed microfluidic chemostats, allowed to examine intrinsic cell dynamics. By adding 19 genes that were not present in JCVI‐syn3.0 cells, they successfully created a new cell called JCVI‐syn3A that was able to undergo normal division [20]. Nonetheless, among these 19 genes, only two are known cell division genes, with all others remaining unclear. So, why these genes are necessary for normal division is still an open question.

- Hack living cells: Aiming at complex eukaryotes

After Venter’s original prokaryotic cells, some researchers turned to the more complex eukaryotes. Starting from the haploid baker’s yeast (*Saccharomyces cerevisiae*) cell that contains 16 chromosomes, followed by eliminating the redundant centromeres and telomeres of the chromosomes via the CRISPR‐Cas9 genome editing technique, Qin and his collaborators in 2018 successfully fused those chromosomes end‐to‐end to create a single‐chromosome eukaryotic cell, the first of its kind in the world [21]. This work clearly demonstrates that even complex life forms can be largely altered through artificial interventions, still performing complex life functions.

- Reassemble components from living cells

In 2022, researchers were able to create an “artificial cell” by adding live bacteria to a synthetic vesicle, followed by the in situ lysis of the bacteria using cell wall hydrolase and the addition of histone and carboxymethyl dextran as nutrients and energy sources [3]. This process involves the disintegration and the automatic reassembly of living cells within vesicles, offering a new idea for constructing artificial cells, that is, using prokaryotes as a source of structural and functional materials. Nevertheless, this artificial cell is not yet capable of self‐replication, which may be due to issues with the synthetic vesicle, suggesting that the boundary of the cell plays a crucial role not only in isolating it from the environment but also in its replication, and this aspect has not yet been fully understood.

### 2.2 Bottom‐up: Integrating well‐defined components

We have listed the essential “components” of life that people proposed in Table 1. Here in this section, we discuss the most cited and critical ones, namely metabolism, genetics, and compartment, by showing their research progress, respectively.

#### 2.2.1 Perspective of metabolism

Prior to the advent of molecular biology, the question of whether life originated from metabolism [22] or genetics [23] was a topic of significant discourse within the scientific community [24]. In the era of molecular biology, the “metabolic‐first” and “genetic/information‐first” have been extensively debated for several decades, still with no clear consensus being reached. Because of the significant gap between the proposed “primordial soup” and “RNA world hypothesis” and the distinct perspectives of both sides of the argument, this debate has been called a “dialogue among the deaf” [24]. We will face huge difficulties if we try to separate “metabolism” and “genetics/information” completely since they are deeply connected. So here we will leave this ambiguity alone, and then call the research line that follows the idea of the Miller‐Urey experiment (i.e., forming a complex chemical reaction network from simple ingredients) the line of metabolism, and call the research line that is from the angle of the double helix structure and the central dogma the line of genetics/information.

Metabolism, originally, refers to the physiological processes that involve the influx and efflux of various substances in living organisms. Yet, the specific meaning of metabolism in pre‐life stages may vary depending on the context. The “metabolic‐first” perspective proposes that the development of an autocatalytic metabolic cycle on primitive Earth was a critical prerequisite for the emergence of genetics. This assertion endeavors to apply fundamental physics principles to biology, with the expectation of discovering general laws that can characterize living systems (e.g., self‐organization and self‐assembly) [24].

For “metabolism‐first”, various theoretical frameworks have been proposed, including Kauffman’s theory of autocatalytic sets [25] (which will be discussed in greater depth in Section 3), Gánti’s Chemoton theory [8], and Maturana and Varela’s theory of autopoiesis [10]. Up to now, there are several critical components developed for these theories: (1) the presence of a multi‐step enzyme‐free chemical reaction system that comprises an autocatalytic set, (2) the capacity for carbon fixation through CO_2_, (3) the requirement for a closed membrane as a defining boundary, and (4) the ability to replicate and to form complex reaction networks [24].

The longstanding criticism of “metabolic‐first” includes whether these metabolic pathways (e.g., autocatalytic networks) are chemically plausible [26] and whether these reaction networks can evolve and be naturally selected [24]. In fact, there are a number of preliminary experimental evidence supporting “metabolic‐first”, including: (1) A series of naturally occurring autocatalytic networks have been experimentally identified, including the formose reaction that generates a mixture of sugars from formaldehyde [27], and the formation of hydrogen cyanide (HCN) tetramers from HCN with formaldehyde presented [28]; (2) The reverse citric acid cycle (also known as the reverse TCA cycle), lying at the core of primitive metabolism, can be established under non‐enzymatic conditions [29]; (3) It has been experimentally demonstrated that early proteins could originate from CO and hydrogen sulfide under the catalytic influence of iron sulfide and nickel sulfide [30, 31, 32].

Besides, recent advancements also began to absorb these challenges further. Lazcano et al. proposed the hypothesis of a semi‐enzymatic origin of metabolism: The emergence of simple metabolic pathways laid the foundation for the key components necessary for primitive organisms [33], and those pathways were initially regulated by non‐enzymes or semi‐enzymes, and subsequently refined through the development of ribozyme‐based and protein‐based pathways [34]. The newly developed software platform “Alchemy” is used to explore and uncover networks of prebiotic chemistry: One such example is that, starting from six simple molecules, namely, water, nitrogen, hydrogen sulfide, ammonia, hydrogen cyanide, and methane, after seven synthetic iterations, an autocatalytic cycle of iminodiacetic acid can emerge (and the experiment has demonstrated that the cycle yield of iminodiacetic acid is 126%) [35]. All of these studies above have partially addressed Orgel’s criticism of whether an autocatalytic set (or network) is merely a mathematical entity that cannot be realized in chemistry and whether these reaction networks can mutate, evolve, and be naturally selected.

#### 2.2.2 Perspective of genetics

Since the idea that life is made up of a series of autocatalytic reactions, Troland believed that the Mendelian factors (or hereditary factors) are enzymes [36], yet RNA had not been discovered. Following this and gradually, scientists started to link RNA to the origin of life [37, 38, 39]. The discovery of two ribozymes triggered the RNA world hypothesis. The RNA world refers to a group of RNA in the early stage of life on Earth that is capable of self‐replication and catalyzing to produce other vital elements of life [40]. DNA and proteins in the central dogma may be late inventions of early life. Although the most primitive forms of life may be based on other molecules instead of RNA, it is now widely believed that all life on Earth could be traced back to RNA [41].

Relics of the RNA world in existing biological systems include nucleotide‐derived coenzymes, self‐processing ribozymes, ribosomes, signaling and messenger molecules—such as adenosine cyclophosphate (cAMP), guanosine tetraphosphate (ppGpp), 5‐amino 4‐imidazole carboxamide riboside 5′‐triphosphate (ZTP), and cyclic dinucleotides—composed of RNA nucleotides or their precursors [42]. The most known relic is the ribosome, a combination of RNA and protein. The structure indicates that the ribosome is a ribozyme, for no amino acid side chain comes within 18 Å of the active site of peptide formation [43]. In addition, the newly discovered aminoacylation from RNA and non‐canonical RNA bases for peptide formation add plausibility to the RNA world [44, 45]. On the other hand, there are many “traces” of the RNA world in the genome. For example, a large number of proteins related to RNA metabolism are found in Last Universal Common Ancestor (LUCA), including modification, polyadenylation, and RNA degradation [46]. Similarly, the core bacterial proteome is closely related to RNA metabolism and RNA‐related information transfer [47].

In conclusion, the RNA world hypothesis is a model for the prebiotic life form on Earth, in which RNA plays a crucial role in the evolution of life. Although this hypothesis fails to provide a perfect scenario of the origin of life, it offers plausible explanations and is supported by independent evidence. It faced two major questions when the RNA world hypothesis was proposed: whether RNA could perform all following‐up functions of life on its own, and whether RNA might have formed on the primitive Earth.

For the first question, researchers found that RNA has the ability to catalyze self‐replication. One type of ribozyme uses the coding of the template to polymerize RNA in the replication step [48], while the other type can contact short RNA sequences together to self‐replicate. After that, a self‐sustained system comprised of two RNA enzymes that catalyze each other’s synthesis was developed [49], which has a doubling time of 1 h and is capable of Darwinian evolution. As RNA that replicates by RNA replicase translated from itself makes RNA‐level Darwinian evolution available, in a 240‐round serial transfer experiment, researchers successfully used the “parasitic” and “host” RNA to form a sustained multiple replicator network with co‐replicative relationships [50].

None of the above experiments considered the origin of RNA on Earth (the *ab initio* synthesis of RNA), that is, the second question. Many hypotheses on the origin of life, based on solving the problem of prebiotic nucleic acid synthesis, consider this to be the most important and necessary step. For this, researchers prefer to start with HCN [51], because as a high energy prebiotic precursor, HCN has the easier generation conditions [52] and could offer adenine or other bases under prebiotic conditions [53]. In addition, HCN chemistry is also considered to have connections with the reductive citric cycle, thus having the potential to form autocatalytic cycles [54]. Based on experiments of HCN and its close relative formamide, researchers such as Saladino et al. demonstrated the plausibility of the prebiotic formamide scenario, which depicts de novo synthesis of RNA strands and metabolic networks [55, 56]. Nevertheless, nucleic acid synthesis involves a number of complex and variable steps and chemical conditions (temperature, pH, catalysts, etc.), including the synthesis of bases and pentose sugar, and the phosphorylation and polymerization of nucleosides. These conditions require careful and delicate designs chronologically, which might be extremely difficult to realize prebiotically [57].

#### 2.2.3 Perspective of compartment

Based on current research, it is believed that the self‐assembling single‐layered micelles or bilayer vesicles by lipids within an aqueous environment are fundamental precursors to the emergence of life (may be referred to as protocells) [58, 59]. Chen and Szostak demonstrated that the simple physical and chemical properties of protocells can give rise to primitive cellular behaviors, such as reproductive competition and energy storage in primitive forms, and that the cooperative interactions between the boundary (namely, the lipid membrane) and the inclusions within these protocells greatly facilitate the transition from holistic replicating reaction networks to true cells [60]. In a recent study, researchers have expanded on the understanding of protocellular complexation by examining how lipids can be chemically modified through endogenous catalytic processes, guiding the transitions from micelles to vesicles and from monomers to biopolymers [61].

It is important to note that the significance of boundaries or compartments encompasses various dimensions. First of all, the compartment is the key to defining individuality. As Gánti highlighted in his Chemoton Theory, while an autocatalytic reaction network can result in an increase in the target substance in solution, it does not necessarily lead to the emergence of “individuals”. It is only when the reaction network is encapsulated by a compartment that the increase in molecular number can be replaced by an increase in the number of individuals [8]. Second, the existence of boundaries is a necessity required by the second law of thermodynamics. As an open system, the living system must be separated from the environment by its boundary, in order to counteract the spontaneous increase of entropy in closed systems [7, 62].

Third, the boundary is often a semi‐permeable membrane, which allows the selective exchange of small molecules and prevents the passage of large molecules, and it thus facilitates certain chemical reactions or dramatically enhances the rate of certain reactions [63]. As in Damer and Deamer’s theory, it was postulated that forming the lipid membrane in water ponds is the kick‐off of the emergence of life, with these membranes featuring many pores that selectively filter incoming and outgoing molecules [64]. Recent research highlights that semi‐permeable compartmentalization may serve as a crucial principle in synthetic cells, as it enables the selective diffusion, retention, and release of structural components, chemical energy, enzymatic substrates, and information input/output [65]. Through this mechanism of permeabilization, cells can communicate with one another, which is a defining feature of living systems [66].

Lastly, the compartment itself might be where the “information” initially arises. The earliest form of information may arise from the molecular composition of the lipids that constitute the compartment, referred to as “compositional information” [67]. Subsequently, more sophisticated carriers of genetic information could emerge, for example, the emergence of polymeric entities such as RNA or DNA [58]. This is the “lipid world hypothesis” proposed by Lancet and Segré et al., even suggesting that the first self‐replicating entity is likely to be single‐layered micelles rather than bilayer vesicles present in modern life, which emerged after micelles [68], because of the benefits of single‐layered micelles, including a heightened sensitivity to variations in composition facilitating more possibilities to form autocatalytic interactions, a high degree of chemical versatility accommodating a wide range of chemical moieties varying in size, geometry, and functional groups, and augmented diffusional interactions, etc. [61, 69, 70]. Furthermore, it has been demonstrated that the amplification of DNA results in the expansion and proliferation of lipid vesicles, thereby establishing a connection between the replication of genetic information and the replication of compartments [71].

## 3 WHAT CAN WE LEARN FROM THE ORIGIN OF LIFE RESEARCH: BUILDING UP COMPLEXITY

Besides the approaches to man‐made life described in Section 2, namely the top‐down approach that aims to minimize complexity by drawing upon existing life forms, and the bottom‐up approach that focuses on integrating well‐defined components, there is another possible approach: Starting with a more abstract definition of life, design and construct the simplest possible systems (from the non‐living and physical/chemical world) that may barely include some basic features of life, and then by iterated design or guided evolution, increasingly complex systems may emerge till they meet the criteria for being considered living. What this route tries to do is actually imitate the gradual prebiotic evolution and the emergence of life from the non‐living world. This idea might be traced back to the Miller‐Urey experiment, in which amino acids (“living substances”) were generated from a variety of small inorganic molecules (“non‐living substances”) through simple physical and chemical processes such as heating, electro‐discharging, condensation, etc. Although the underlying mechanisms were not clear, it seemed that we only needed to prepare for the right substances (which are not very complex) and the right conditions (which are not very sophisticated and delicate), and the transition could occur automatically. It was then speculated that a “magic event” could even occur, leading to the emergence of life from these small molecules, just as what had actually occurred on Earth billions of years before.

While the aforementioned logic may seem straightforward, determining which substances and conditions should be included is a rather complex issue. This made a category of researchers, such as some synthetic biologists, resort to the delicate bottom‐up integration of well‐defined components, as mentioned before, while another category of people turned to non‐chemical or virtual systems, aiming to discover the underlying general mathematical principles governing how basic properties of life, such as self‐replication emerge de novo, without focusing on wet experiments. The latter category branched into a field called “artificial life” (more properly, it should be “soft artificial life”), resulting in developments such as evolutionary algorithms and artificial ecology (see Box 1 for its brief history, as this field has strayed from the main focus of this minireview). The remaining in this category continue to focus on understanding the underlying mathematics of life, with the most notable example being Stuart Kauffman’s work on autocatalytic sets and related studies.

This mathematical line of research is highly relevant to the field of man‐made life, and many of its successes can be applied to the creation of man‐made life (although there is still a big gap between these mathematical theories and the actual protocell creation). However, this approach, which could be called “bottom‐up from the origin of life”, has received relatively little attention from experimental biologists/chemists and synthetic biologists. This may be due to the fact that this approach often only uses simple and small molecules that do not necessarily require sophisticated synthesis, yet rather emphasizes the building and coupling of chemical reaction networks. Here in this section, we focus on discussing this “bottom‐up from the origin of life” approach, starting from the autocatalytic sets theory. The advantages of this approach are discussed, as well as the challenges it faces in creating a man‐made life, for example, the issues on the compartment and the ability for evolution. Then, in subsequent subsections, we will discuss potential solutions to these challenges.

3.1

**BOX 1 A brief history of the research field of “soft artificial life” (often abbreviated “ALife”).**

In the late 1940s, John von Neumann delivered a lecture entitled “The General and Logical Theory of Automata”, in which he proposed the concept of an “automaton” as a machine that is capable of performing actions logically and sequentially based on a combination of the external information from its environment and its internal state. He further posited that it would ultimately be discovered that natural organisms also follow similar principles [72]. This idea might be the seminal concept of “artificial life”, although not the name yet.

Afterward, many scientists directed their attention toward this field, resulting in a number of noteworthy contributions. In particular, in the 1960s, John Conway developed one of the most renowned examples of cellular automata, referred to as the “Game of Life” [73]. It is a two‐dimensional grid of “cells”, and the state of a cell is updated based on the state of the surrounding cells. Despite its simplicity, this fully deterministic and local updating rule results in rich dynamics and emergent behaviors. Game of Life has been proven to be Turing complete, meaning that it can simulate any other Turing machine. In the 1970s and 1980s, a significant amount of research was devoted to this field. In the 1980s, Christopher Langton simplified the 29‐state automaton proposed by John von Neumann to an 8‐state variant, based on the concept of cellular automata, and successfully created the first self‐replicating computer organism [74]. Further, Stephen Wolfram created the simplest version, called the elementary cellular automata, and comprehensively investigated and classified its various behaviors, also demonstrating their potential application to natural phenomena, published in a notable book “*A New Kind of Science*” [75]. Currently, models of cellular automata not only serve as exemplary demonstrations of emergent behavior but also continue to play a vital role in various areas of research, such as modeling wound healing [76], simulating quantum lattice gases [77], quantum dot cellular automaton [78].

In 1987, Christopher Langton, the scientist mentioned above at the Santa Fe Institute, formally coined the term “artificial life”, ALife for short [79]. His definition of ALife can be succinctly summarized as the study of the artificial system in virtual environments such as computer simulations, which exhibits the characteristic behaviors living systems have, in order to understand the underlying information process mechanism that defines such systems. In general, the methods and techniques developed in the field of ALife include cellular automata (as mentioned above), agent‐based (or program‐based) models, evolutionary algorithms, etc. Since this term, and with the rapid advancement of computer technology, ALife has undergone significant expansion and development. It deserves to mention that ALife not only has an impact on science but also on popular culture, including a number of video games that follow the idea and concepts of ALife, such as Creatures (1996 [80]), Spore (2008), Darwinists (2014), and Species (2018).

For agent‐based models, Craig Reynolds created a program in 1987 that successfully reproduced the behaviors of a flock of birds with three straightforward locally updating rules [81], thereby initiating a sequence of subsequent studies on collective animal behavior, such as the notable Viscek model [82]. In 1991, Tierra was developed to simulate biological evolution. It simulates the dynamics of competition and cooperation among organisms, each of which is referred to as an “agent” and represented by a computer program, and their ability to adapt to environmental changes [83]. Later, Tierra inspired Charles Ofria, Chris Adami, et al., to develop Avida, which is still under active development. The agents in Avida are able to acquire resources that are utilized to self‐replicate by executing elementary computational tasks written in their “genome”, thus enabling the transfer of an agent’s genome to the next generation. Through this mechanism, Avida is capable of simulating the evolutionary process [84].

The evolutionary algorithm is a useful and practical tool developed in the field of ALife, yielding high‐quality solutions to optimization and search problems [85]. The basic idea behind evolutionary algorithms is the initiation of a population of solutions (encoded as a vector of integers, for example) and the subsequent evolution over a series of generations through a set of rules inspired by natural evolutionary processes, such as selection, crossover, and mutation. One of the most renowned evolutionary algorithms is the genetic algorithm, which was first proposed by John Holland. Genetic algorithm has been extensively applied in various practical domains, including industrial design, circuit design, production scheduling, and so forth [86, 87].

More recently, in the field of ALife, an international open science project OpenWorm was developed, ultimately aiming to model and simulate all 959 cells of the worm *Caenorhabditis elegans*, thus its entire set of behaviors. If successful, it would be the first time that people fully characterize the behavior of an entire organism [88]. Another project Neurokernel, based on similar ideas, aims to build an open‐sourced platform to simulate the functionality of the whole brain of the fruit fly *Drosophila melanogaster*. It comprises a set of libraries and tools that facilitate researchers in constructing and executing brain models on multiprocessor systems [89].

### 3.2 Bottom‐up from the origin of life: Autocatalytic sets

A lot of literature above has emphasized that metabolism must be an indispensable component of life (if not the only indispensable one), no matter man‐made or natural life. Metabolism, in the contemporary sense, refers to the whole set of chemical reactions that sustain the living of a cell or an individual. Yet, upon the very beginning of natural life or man‐made life, a well‐defined individual may not yet exist [90, 91], and thus, metabolism was simply the collective term for all chemical reactions within the system that facilitate various functions such as self‐replication, one of the key characteristics of life [92, 93, 94]. So, the crucial question arises: What chemical reactions must be included in the system, and how should they be coupled in order to allow the entire system to perform functions such as self‐replication found in living life?

In 1993, Stuart Kauffman proposed that, generally speaking, a chemical reaction system can exhibit self‐replication if the products of one reaction in the system act as catalysts for another reaction in the same system (i.e., the system becomes closed). That is, the amount of all substances in that system can increase exponentially given sufficient raw materials, resulting in self‐replication at the system level. He referred to this system as an autocatalytic set and suggested that the emergence of life may have originated from such sets [25, 95]. Steel and Hordijk et al. further developed the autocatalytic set theory into a more mathematically comprehensive theory called reflexively autocatalytic and food‐generated (RAF) theory [96].

**FIGURE 2:**
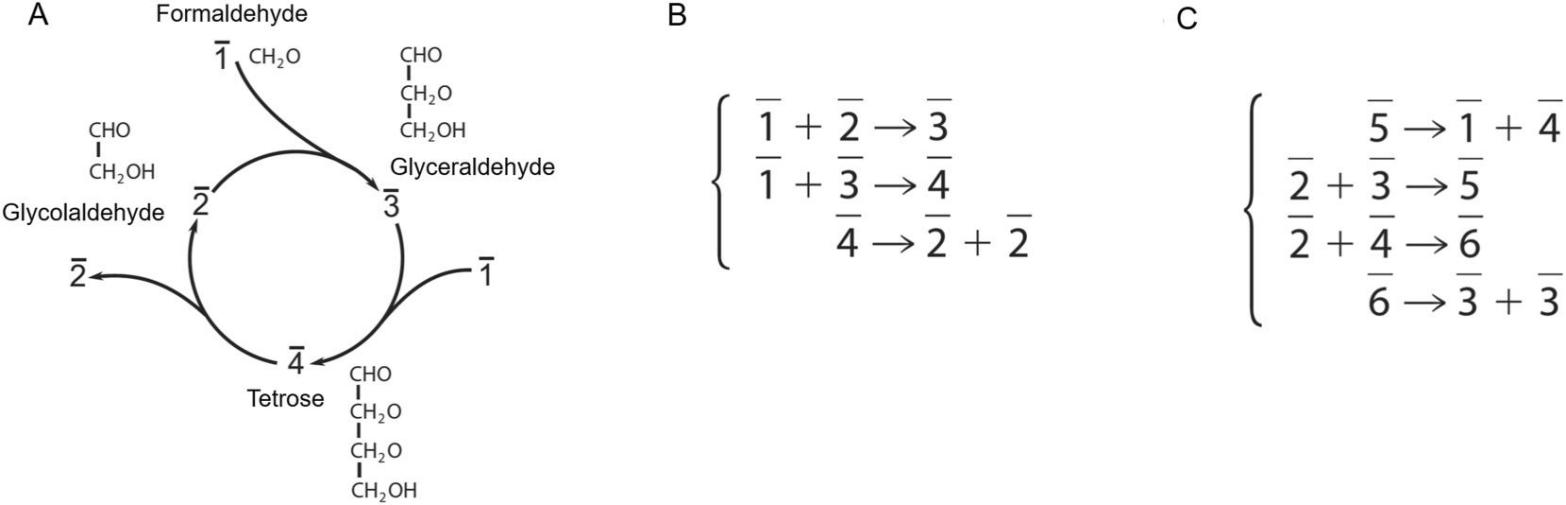
Examples of chemical reaction systems that are able to self‐replicate. Reproduced with the permission of ref. [92]. Copyright © 2018 Liu and Sumpter. (A) A simple representation of the formose reaction. (B) Another representation of the formose reaction, represented by reaction equations. (C) Another imaginary self‐replicating reaction network. We can check that all these systems satisfy the three criteria above.

In fact, further studies have revealed a relaxed condition for self‐replication, that is, it is not necessarily required, as in RAF theory, that every reaction in the system is catalytic [92], because any catalytic reaction can be mathematically rewritten as a set of coupled non‐catalytic reactions. Here, we summarize the relaxed conditions: If in a chemical reaction system,

1. For each reaction, at least one type of its reactants comes from the products of other reactions (called the criterion for self‐driven).
2. There are some types of intermediate molecules, and the number of times they appear on the reactant side is less than that on the product side (called the criterion for overproduction).
3. There is no type of intermediate molecule for which the number of times it appears on the reactant side is larger than that on the product side (called the criterion for no‐overtake).

then this chemical reaction system must be able to self‐replicate. But it deserves to mention that if only the first two criteria of self‐driven and overproduction are satisfied, the system is very likely to be able to self‐replicate. Several examples from [92] are provided below (additional examples can be found in the supplementary information of refs. [92, 97]):

The system in Figure 2A,B is a simple representation of the formose reaction, which would be the simplest example of a self‐replicating reaction network, where $\bar{1}$ stands for formaldehyde, $\bar{2}$ for glycolaldehyde, $\bar{3}$ for glyceraldehyde, and $\bar{4}$ for tetrose, respectively. First of all, $\bar{2}$ in the first reaction comes from the products of the third reaction, $\bar{3}$ in the second reaction comes from the products of the first reaction, and $\bar{4}$ in the third reaction comes from the products of the second reaction, so the criterion for self‐driven is satisfied. Second, $\bar{2}$ appears twice on the products side while once on the reactant side, so the criterion for overproduction is satisfied. Lastly, no molecule appears more often on the reactant side, so the criterion for no‐overtake is satisfied. Likewise, we can verify the system in Figure 2C is also self‐replicating.

Self‐replication is well‐defined theoretically, as mentioned, but it can be challenging to be identified experimentally due to the fact that the definition relies on a comprehensive understanding of all chemical reactions in the system, yet it is often difficult or even impossible to fully identify all relevant reactions [98]. We thus proposed the criteria in ref. [98] that allow for the experimental determination of whether a chemical reaction system is self‐replicating or not (note that self‐replicating and self‐sustaining systems are often equivalent in terms of network topology, and can be converted from one to the other under different conditions): A chemical system that is in a continuous‐flow stirred tank reactor (CSTR) and subjected to an inflow *f* is self‐sustaining if and only if the following two criteria are satisfied (Table 2): After the transient period, (1) for a non‐zero initial condition *ξ*, the outflow *f′* is distinct from the inflow *f*, and (2) for a zero initial condition *ξ*_0_, the outflow is identical to the inflow *f*. Here, “zero initial condition” means that there is nothing in the tank reactor at the beginning of the experiment (otherwise “non‐zero initial condition”), referring to ref. [98] for details. According to the criteria, to experimentally determine whether a system is self‐sustaining, we only need to compare the outflow and the inflow of the CSTR.

**TABLE 2:** The criteria for a chemical system to be self‐sustaining.

(i)	$f_{\varepsilon ,f}^{\prime }=f$	$f_{\varepsilon ,f}^{\prime }\neq f$	$f_{\varepsilon ,f}^{\prime }=f$	$f_{\varepsilon ,f}^{\prime }\neq f$	
(ii)	$f_{\varepsilon_{0},f}^{\prime }=f$	$f_{\varepsilon_{0},f}^{\prime }=f$	$f_{\varepsilon_{0},f}^{\prime }\neq f$	$f_{\varepsilon_{0},f}^{\prime }\neq f$	
				$f_{\varepsilon ,f}^{\prime }=f_{\varepsilon_{0},f}^{\prime }$	$f_{\varepsilon ,f}^{\prime }\neq f_{\varepsilon_{0},f}^{\prime }$
	Trivial	Self‐sustaining	Impossible	Sequential	Sequential + self‐sustaining

*Note*: Here *f′* is the outflow, and *f* is the inflow. *ξ* represents a non‐zero initial condition, while *ξ*_0_ represents the zero initial condition. Reproduced with the permission of ref. [98]. Copyright © 2020, The Author(s).

In addition to autocatalytic sets and RAF theory, there are two similar ideas. In 1994, Fontana proposed the concept of *λ*‐calculus, which involves viewing a molecule as a mathematical function and its interaction with another molecule as a functional operation, that is, these functional operations are considered the logical equivalents of chemical reactions [99]. This theory is more ambitious and mathematically general, but also difficult to use in practice. In fact, it reaches similar conclusions about self‐replication as RAF theory. Another theory is the chemical organization theory (COT) proposed by Dittrich and Banzhaf et al., as an alternative formal framework for studying the origin of life [100]. It is based on set theory and defines a closed and self‐sustaining set of components (rather than a network as in RAF theory) as a chemical organization. In COT, it also does not require that every reaction is a catalytic reaction [101].

There is substantial experimental evidence supporting RAF theory and the related ones mentioned above. For example, the entire metabolic network of *Escherichia coli* has been observed to be a giant autocatalytic set [102]. By using small organic molecules such as certain thioesters and sulfides, Semenov et al. successfully constructed a reaction network exhibiting autocatalytic, bistable, and oscillatory behavior, suggesting that the complex regulatory dynamics associated with cellular function may have emerged spontaneously before the emergence of cellular life [103]. Otto et al. successfully designed and constructed a self‐replicative chemical system, in which basic blocks (a small peptide‐containing molecule) can join together to form cyclic trimers, tetramers, and hexamers, with the production of hexamers dependent on the presence of trimers and tetramers. The hexamers can stack together and breaks when long enough, whereas the broken remnants can continue the stacking process, thus exhibiting the self‐replicating behavior [104, 105]. Nanda et al. analyzed the complex reaction network in a β‐sheet peptide system and found that the network is not only structurally stable and autocatalytic, but also error‐correcting and evolvable [106]. Recently, Peng et al. showed that two massive reaction networks are hierarchically organized autocatalytic [107]: one includes all the reactions that are relevant to the origin of life in the Kyoto Encyclopedia of Genes and Genomes (KEGG) reaction database [108], and the other includes radiolytic and geochemical reactions [109].

In conclusion, for the task of man‐made life, the lesson we can learn from the autocatalytic sets theory, RAF theory, and related works is that: We can purposefully design systems that meet these mathematical criteria, with which self‐replication, a main feature for “metabolism”, would be automatically granted. This realizes the first step toward a living system (in fact, there have been successful examples, as mentioned before [44, 45]). Nevertheless, although we can design certain chemical reaction networks in which some molecules are able to replicate, we cannot call this type of molecule or the entire reaction network life, as it faces at least two major concerns: (1) how individuality is defined in these systems (or equivalently, how the compartment separates the system from its environment), and (2) the ability to undergo evolution. In the following two subsections, we will discuss the two concerns and try to propose potential solutions.

### 3.3 What about compartment/individuality: Alkaline hydrothermal system

Autocatalytic sets theory (or RAF theory) does not emphasize the concept of compartment or individuality. It posits that the molecules that constitute the compartment should emerge through the self‐replicating system (namely, the autocatalytic network) together with other molecules in the network. Thus, it is not necessary to consider compartment molecules exclusively. However, in practical implementation, how to design a boundary system that is able to self‐replicate and integrate it into the metabolic network of the “protocell” is still an open question. In this section, we approach this question in an alternative way: Is it possible that the boundary of the primordial cell was solely a physical boundary, and that over time, the cell gradually “invented” the boundary molecules so that it becomes free‐living like the modern cells? To start with, we provide an overview of relevant theories, from Günter Wächtershäuser’s iron‐sulfur world to William Martin and Michael Russell’s theory on alkaline hydrothermal vents.

In the 1980s, Wächtershäuser put forward the “iron‐sulfide world” as a theory for the pre‐biochemical evolution. It systematically traces back the origins of current biochemical processes to primitive reactions and suggests that life may first be formed on the surface of iron sulfide minerals [30]. In contrast to the classic Miller‐Urey experiment, which requires external energy sources such as simulated lightning and heating, Wächtershäuser’s system incorporates an intrinsic energy source derived from iron sulfides (e.g., pyrite) and other minerals: The energy generated by the redox reactions of these metal sulfides could be harnessed to synthesize organic molecules, and this system may have evolved into an autocatalytic set, a self‐replicating and metabolically active entity that predates extant life forms [30, 110]. This theory is premised on the idea that the surface of iron sulfides which are positively charged, may have enabled the polymerization through the adhesion of reactants and their proper alignment with one another [30].

This theory encompasses several key points. First of all, it was suggested that the particular place of the origin of life might be in a volcanic hydrothermal vent at high pressure and high temperature under the deep sea, which was first thought to be the black smoker [111, 112]. These hydrothermal vents provide not only a constant source of raw materials and chemical energy released by redox reactions, for example, between hydrothermal hydrogen and carbon dioxide, but also a steep temperature gradient that enables partial reactions to take place at the optimal locations within the vent [113, 114]. Second, this theory suggests that the early chemistry of life occurs not in 3‐dimensional bulk solutions but on 2‐dimensional mineral surfaces such as iron sulfide. It thus emphasizes the crucial role played by surfaces, including but not limited to: (1) The rates of certain reactions are dramatically increased if occurred on surfaces than in bulk solutions due to the high selectivity effect of the surface [30]; (2) Surfaces of these hydrothermal vents can provide a number of transition metal centers (predominantly iron and nickel) as catalysts for carbon fixation pathways [115, 116, 117, 118]; (3) These carbon fixation pathways produce small organic molecules that act as organic ligands of the metal centers, from inorganic gases, gradually forming autocatalytic reaction networks (e.g., the proposed reductive citric acid cycle, in particular) [113, 114]. This set of processes is referred to as “surface metabolism” [30]. Although this theory emphasized the significance of the 2‐dimensional boundary, it did not address how the compartment/boundary that enables the free‐living of cells comes about.

Later, as Martin and Russell suggested [113, 114, 119], it was realized that the black smoker type of hydrothermal vent might be too hot (*ca*. 350°C); instead, the first life form might have evolved inside the alkaline hydrothermal vent (*ca*. 100°C) in the deep sea (the Lost City vent system, first discovered in 2000, serves as an example of such a location [120, 121]). These alkaline hydrothermal vents comprise a great number of microscale caverns coated by thin membraneous metal sulfide walls. This theory is able to address the problem of the emergence of compartments, which was missing in Wächtershäuser’s original theory [113, 122].

1. While amphiphilic molecules can spontaneously self‐assemble into micelles or vesicles, achieving the division into two is still challenging and may necessitate a highly intricate mechanism. The alkaline hydrothermal vent theory instead postulates that the earliest cells were not free‐living but had membrane‐like physical boundaries, namely, those microscale caverns. Further evolution leads to self‐supporting lipid membranes and closed cells, thereby leaving the microcavern and becoming free‐living. This is supported by the fact that bacteria (plus eukaryotes) and archaea have entirely different types of membrane lipids, suggesting that they emerged independently.
2. The microcaverns help concentrate molecules that are newly synthesized, increasing the chance of forming oligomers or polymers.
3. The synthesis of lipids that make up the boundary of a cell to separate it from the external environment is not indispensable until almost all cellular functions are ready.

In a sense, a compartment in the alkaline hydrothermal vent can be considered a “protocell”. In fact, if not constantly immersed in an aqueous environment (e.g., in alkaline hydrothermal vents), the formation of “protocells” might be even more accessible because it has been demonstrated that wet‐dry cycles and exposure to ultraviolet (UV) light can enhance the formation of membranes from lipids and lead to the synthesis of more varieties of biomolecules [123, 124, 125]. Another “clay hypothesis”—clay minerals are hydrous aluminum phyllosilicates (e.g., kaolinite and montmorillonite), made up of crystals—that was popularized in the 1980s while first developed in the mid‐1960s by Graham Cairns‐Smith [126], even postulates that the clay crystal itself, which is more like to be the boundary and obviously non‐living, is the individual that is naturally selected for in the first place, and later, after complex organic molecules that are catalyzed by the surface of the clay crystals perform a “genetic takeover” from their clay “vehicle”, those molecular systems become the individuals that are subject to selection [127, 128, 129].

Above all, both the iron‐sulfur world hypothesis and Russell’s theory placed a significant emphasis on the role of the physical surface [113, 114, 119, 126]. Thus, it may be prudent to adopt an alternative approach when creating a man‐made life: Instead of initially focusing on designing and integrating independent boundaries, it may be more beneficial first to establish autocatalytic reaction networks confined by physical boundaries and then anticipate the gradual development of independent boundaries for free‐living, through evolution. Evidently, this approach is akin to a simulation and realization of the origin of life. Although there is currently limited experimental research following this idea, there have been some studies that may provide valuable insights. For example, the research conducted by Vincent and Baum et al., through a serial transfer experiment on a mixture of a synthetic “prebiotic soup” and mineral grains, has demonstrated the potential for the spontaneous emergence and the enrichment of self‐replicating mineral‐associated autocatalytic systems [130, 131]. In a simulated chemical space developed by Wołos and Grzybowski et al., as having been briefly mentioned above, it was observed that surfactant molecules (such as straight‐chain saturated fatty acids and *α*‐hydroxy acid surfactants) could be generated naturally as a result of multiple reactions of aldehyde homologation, which implies that primitive cell compartments, or vesicle structures, could have emerged spontaneously in early Earth environments, starting from simple molecules [35]. Recently, researchers created an inorganic hollow colloid (replacing lipids, as an alternative option for the compartment) with a well‐defined micropore on its surface, which was able to perform active transport, thus an inorganic cell‐mimics [132].

### 3.4 How to evolve from simple to complex: Ladderpath system

As mentioned, we anticipate that starting from relatively simple autocatalytic reaction networks confined by physical boundaries, subsequent evolutionary processes would allow the formation of complex molecules, including lipids, for example, that can serve as the ingredients for independent boundaries. Yet, is this logic backed up by any theory? In fact, many studies above have already implied such logic that there is a natural tendency to evolve from simple to complex. For instance, the iron‐sulfur world hypothesis implies that once a primordial autocatalytic network was established on the surface, there would be a gradual emergence of more complex autocatalytic networks and complex organic compounds, even the information molecules [30]. In RAF theory, the RAF sets at a preceding level will serve as the raw material for the RAF sets at the subsequent level, thereby facilitating the formation of increasingly complex networks [96]. The theory of seed‐dependent autocatalytic system proposed by Peng and Baum et al. suggests that the prebiotic Earth may have undergone a gradual complexation by successive triggers of different autocatalytic systems (not through raw materials as posited in RAF theory, but through “seeds”) [107]. Nonetheless, is there a general theoretical foundation for the aforementioned arguments, that is, what are the necessary conditions for a system to evolve from a simple to a complex state (obviously, not all systems possess such characteristics)?

**FIGURE 3:**
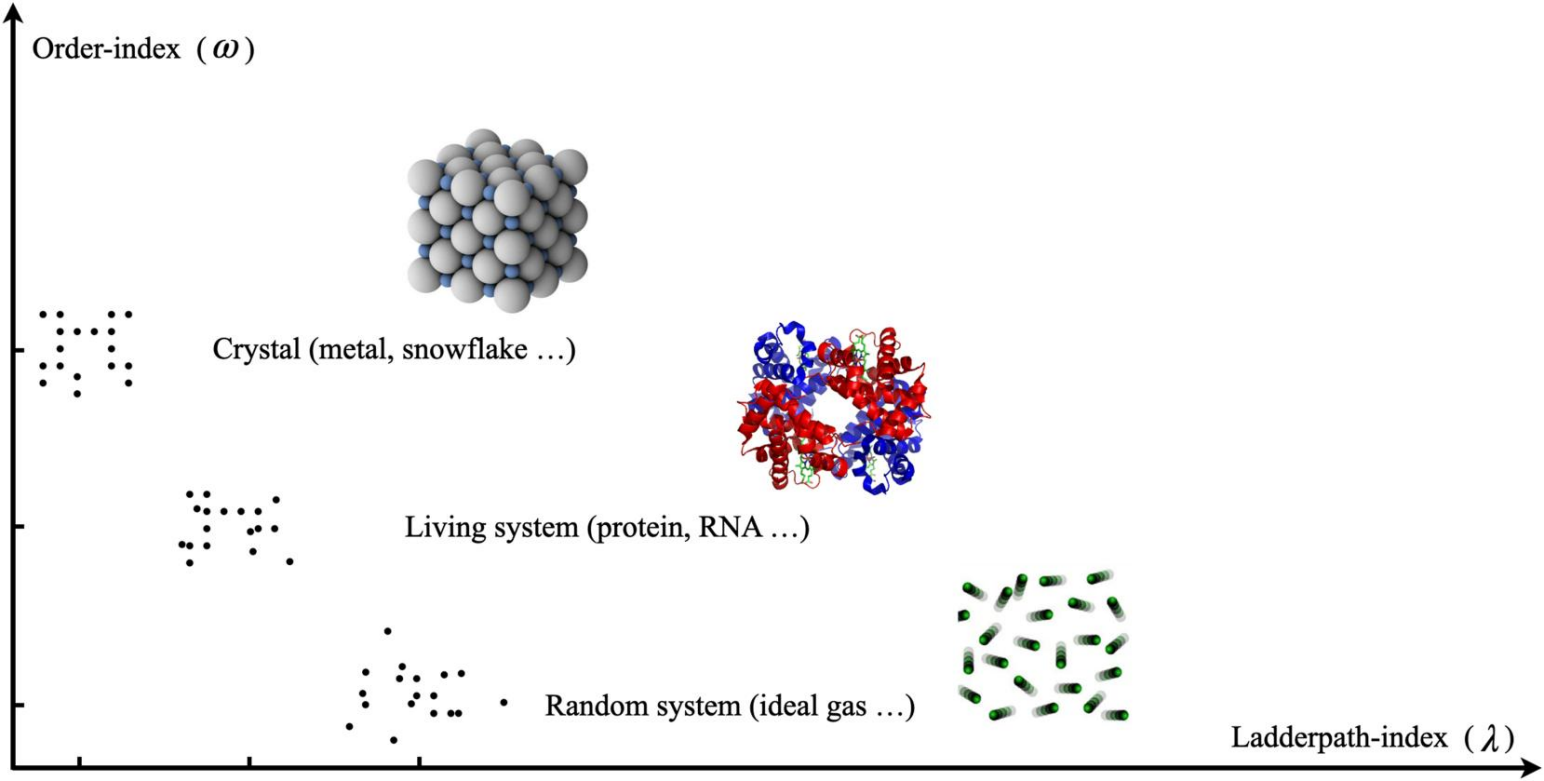
Conceptual sketch of the two aspects of complexity, suggested by the ladderpath theory.

First of all, it is essential to clarify the concept of “complexity” which is often ambiguous, particularly in the context of evolution [133, 134]. Let us consider a seemingly paradoxical example: In our intuition, the complexity of protein molecules is evidently higher than that of an ideal gas consisting of random‐moving molecules, due to the higher level of organization present in the former; in the meanwhile, intuition also suggests that the complexity of proteins is higher than crystals which are even more highly ordered. We thus face a dilemma: Why, in the case of ideal gases, a higher degree of order equates to greater complexity, while in the case of crystals, the opposite makes more sense intuitively? Recently, we proposed the “ladderpath theory”, suggesting that our intuitive concept of “complexity” has two distinct aspects measured by the ladderpath‐index (*λ*) and the order‐index (*ω*), respectively. The former, *λ*, quantitatively measures the cost and difficulty of constructing a target system, while the latter, *ω*, quantifies the degree of organization and hierarchical structures exhibited in the target system. Complexity can then be quantified by combining *λ* and *ω* in a certain way, for example, taking the product of the two [135, 136]. As shown in Figure 3, an ideal gas system and a crystal are two representative extreme cases: The former is close to the *x*‐axis, indicating high *λ* but low *ω*, and the latter is close to the *y*‐axis, indicating high *ω* but low *λ* (referring to ref. [136] for how to calculate the indices of the exemplified black dot patterns); while living systems have both high *λ* and high *ω*, resulting in high complexity.

The Ladderpath theory further (1) proposes the definition of the “ladderpath‐system”, that is, a system is a ladderpath‐system if it satisfies the following two criteria.

**FIGURE 4:**
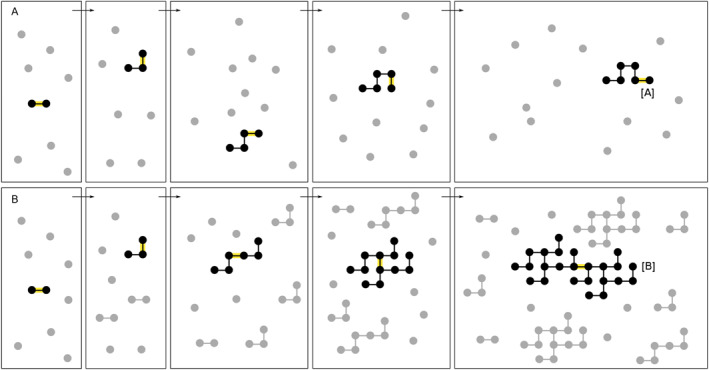
(A) The evolution of a system that is not a ladderpath‐system. (B) The evolution of a ladderpath‐system. The box represents the system (its size does not matter). The system evolves from left to right. Every new combination (i.e., the way to generate new structures) can be considered as, for example, forming a new chemical bond between two atoms, highlighted yellow. The newly generated structures are shown in black, while already‐existing structures are shown in gray (which is only for the reader to recognize and irrelevant to the dynamics).

- It is able to generate new building blocks (named “nucleation”);
- Some building blocks are able to replicate (named “replication”).

and (2) shows that the complexity of a ladderpath‐system has a natural tendency to increase. To illustrate this, consider a highly simplified ideal experiment. As shown in Figure 4A, we assume that the system has the ability to generate new building blocks or structures (represented by the black and gray dots), but that these structures cannot replicate (so this system is not a ladderpath‐system). Initially, there are many basic structures, namely, the single dots. The structure [A] can be generated in five successive combinations. Next, consider another system in Figure 4B, assuming that it can also generate new structures but that these structures can replicate (i.e., a ladderpath‐system). We can see that the structure [B] can be generated in five steps. Based on the ladderpath theory, we can calculate the complexity of [A] and [B]: (1) The ladderpath‐index (*λ*) of [A] and [B] are both 5, as they both require five steps; (2) Based on the definition of order‐index *ω*:= *S* − *λ* (where *S* is the size‐index, namely, the total number of constituent black dots in this case), the order‐index (*ω*) of [B] is 24−5 = 19 where 24 is the size‐index of [B], while *ω* of [A] is 6−5 = 1; (3) Thus, the complexity of [B] is 19×5, while that of [A] is 1×5, which is smaller than the former. This conclusion that [B] is more complex than [A] is consistent with our intuition. Thus, in general, we can say that it is easier and takes fewer steps to generate larger and more complex structures in a ladderpath‐system than in other systems.

Therefore, our anticipations are grounded because (1) autocatalytic sets possess information in a broad sense, that is, the information carrier and the context are not completely separable, and (2) an autocatalytic set fulfills the two criteria of ladderpath‐system, thus exhibiting a tendency of the natural increase of complexity, and a gradual accumulation of the information it contains. Yet, how the information accumulates is contingent upon the methods employed in the particular human‐directed evolution. Still, as long as we require the presence (or emergence) of self‐replicating systems at each iteration in the directed evolution, we can reasonably anticipate that—starting from the preliminary information in the broad sense, through the gradual formation of more intricate networks and molecules—certain molecules capable of storing information are able to emerge ultimately (i.e., the emergence of the information in the narrow sense). The three examples of anticipated complexity increase mentioned at the beginning of this section can also be backed up by the ladderpath theory (although this tendency was taken for granted in those works), because the systems in the three examples fulfill the criteria of nucleation and replication, and thus are all ladderpath‐systems.

The final remark is that although here we expect genetics and the compartment can ultimately emerge from metabolism (in particular, autocatalytic sets), thereby forming the life form as we know, any pathway that is able to integrate the three factors is an option. In the history of Earth, this type of integration might have occurred more than once. Under a similar primordial physicochemical condition simulated in the lab, it is very likely that an alternative evolutionary path would be taken, referred to as the “multiple realizations of life”. Indeed, the path to synthetic life need not mirror the historical path on Earth, as the latter was under specific conditions and subject to contingencies. In synthetic biology, we can use the generic path to guide the design of man‐made life. By comparing the particular needs with the generic path, combined with accessible tools, there is a high probability that the researchers will find the most feasible solution.

## DISCUSSION AND CONCLUSION

Before summarizing what we did in this review, we use two paragraphs to discuss and emphasize the goal of man‐made life research, to better motivate this work. On a conceptual level, the ultimate question of man‐made life is reproducing the process of how “information” emerges from “nothing”, theoretically and empirically. Yet, first of all, it is essential to clarify what the word “information” here refers to. The broader and more general perspective is that “information” can be defined as a reduction of uncertainty (namely, information entropy), reflected as a change in the probability distribution. This view is primarily held by physicists and is rooted in information theory. For example, in an autocatalytic reaction system, a seed molecule can trigger a sub‐system, changing the composition of the entire system, which in turn affects the evolution of the future system, thereby modifying the probability distribution of subsequent events. In this sense, the autocatalytic system possesses information. Therefore, it has been proposed that autocatalytic sets have “preliminary heredity” [98]. Besides, in the “lipid world” hypothesis, the molecular composition of lipids is also defined as “compositional information” [67]. The other school of thought on information is more specific, stating that the information carrier and the content must be separated and independent [8]. For example, the information encoded in a fiction book is the story and plot, etc., having nothing to do with the information carrier, for example, what type of paper the book is painted on and what kind of ink is used. For life on Earth, this essentially refers to the genetic information of organisms carried by and encoded in linear polymers such as DNA and RNA, with the direction of flow governed by the central dogma [137]. For man‐made life, the information carrier could be different from DNA/RNA, but we just have not found the alternative yet.

In fact, the ultimate goal of man‐made life research is a realization from null information, to information in a broad sense, to information in a narrow sense. The emphasis is on the second “to” since the emergence of broad information is relatively easy, as exemplified by the numerous instances of autocatalytic sets that exist. Szathmáry once suggested that the origin of life is characterized by three stages: from holistic replicators, to replicators with limited hereditary information, to replicators with unlimited hereditary information. Here “holistic” refers to broad information, while the latter two refer to narrow information (limited heredity refers to RNA and unlimited heredity refers to DNA) [138]. In any case, when the transition from broad information to narrow information is realized, the goal of man‐made life research is achieved. Only then is the concept of multiple realizations of life understood, and the underlying mechanism behind the origin of life answered (not just the historical problem of the origin of life on Earth).

So, in this paper, we first reviewed the two main approaches often employed in the field of man‐made life: the top‐down approach that reduces the complexity of extant and existing living systems (briefly described in Section 2.1) and the bottom‐up approach that integrates well‐defined components (elaborated in Section 2.2). We can see that neither of them is sufficient to address these challenges in this field because they both pre‐set the existence of independent information carriers, that is, narrow information pre‐exists. Thus, in Section 3, we argued for another possibility that can also be categorized as “bottom‐up”, but from a more primitive level, the origin of life: Starting with the construction of autocatalytic chemical reaction networks that employ physical boundaries as the compartments initially, then designing directed evolutionary systems, with the anticipation of the eventual emergence of independent compartments so that the system becomes free‐living (this anticipation is backed up by a newly developed theory, the ladderpath theory, summarized in Section 3.3). This approach is actually analogous to the process of how life originated. After decades of research into the origin of life, many quantitative mathematical models and tools have been developed, yet to be applied to the vein of man‐made life. Through this paper, we hope to draw the attention of synthetic biologists and experimentalists to a more theoretical perspective, and promote the communication between the origin of life community and the man‐made life community.

Moreover, it is worth mentioning some technologies that have great potential for creating man‐made life. For example, microfluidics is used to integrate well‐defined components [17]: Deshpande et al. used microfluidic chips to inject their chosen proteins into lipid vesicles, named octanol‐assisted liposome assembly [139]; Weiss et al. used microfluidics to “upload” ATP synthase into vesicles, resulting in rudimentary mitochondria capable of making ATP inside the vesicles [140]. For software, the program “CatlyNet” has been developed to calculate RAFs (loosely speaking, autocatalytic sets) and other sets from any set of reactions and foods [108, 141]. Unlike the idea of retrosynthesis (although retrosynthesis includes a lot of software such as Chematica and RetroPath2.0 [142, 143]), the aforementioned “Alchemy” platform has been developed to explore synthetic pathways for biotic molecules from user‐defined starting ingredients, allowing researchers to design possible prebiotic evolution from simple molecules to complex macromolecules [35]. Not only is it possible to identify pathways in software, but the recent development of automated chemistry can assist researchers in the synthesis of various molecules, which can save a lot of effort when diverse molecules are demanded in the constant attempt when creating man‐made life, such as the work of Burger et al. who developed an automated synthesis robot that performed 688 consecutive reactions in 8 days and discovered a photocatalyst for hydrogen production from water [144], and the Chemputer system that has automatically synthesized three pharmaceutical compounds in a few days, with yields comparable to those reported in previous literature [145]. Besides, the idea of directed evolution can also be employed, applying the iteration of mutation‐selection‐accumulation cycles and the serial transfer technique. Recent developments include machine learning that could screen and re‐pick discarded information from failed trials, thereby increasing effectiveness [146, 147]. Lastly, note that relevant technologies include, but are not limited to, those mentioned above.

In synthetic biology, the attempt to integrate metabolism, genetics, and compartments, by using pre‐existing substances, or the design of genetic circuits may not provide a comprehensive explanation of the origin of life. On the other hand, if the origin of life research does not address the transition from broad to narrow information, it will have limited help in the “multiple realizations of life” or creating a man‐made life. In the short term, understanding the emergence of narrow information (i.e., translation or the replication catalyzed by enzymes) from autocatalytic reaction networks is a crucial problem faced in front of both fields. In the long term, the real comprehension and reconstruction of living systems may require the actual initiation of autocatalytic networks from primordial molecules, with the gradual emergence of new functions until a protocell is fully formed. Only then is the possibility of the emergence of living systems from the physical‐chemical realm through evolution fully demonstrated. Indeed, the full reconstruction is crucial for identifying extraterrestrial alien life in astrobiology, as well as for the initiation of proto‐life and evolution in an environment that is distinct from Earth (even in a virtual environment). In turn, by reconstruction, we will be able to see the full range of possibilities for life on Earth, from its components to its organization and, ultimately, the entire system. Only then will we be able to fully recognize, design, and recreate life. The success may not come at a single moment but through a long and increasingly exciting period.

## AUTHOR CONTRIBUTIONS

**Lu Peng**: Formal Analysis; Investigation; Validation; Visualization; Writing – original draft; Writing – review & editing. **Zecheng Zhang**: Investigation; Methodology; Writing – original draft; Writing – review & editing. **Xianyi Wang**: Investigation; Validation. **Weiyi Qiu**: Conceptualization; Investigation; Methodology; Writing – original draft; Writing – review & editing. **Liqian Zhou**: Funding acquisition; Resources; Writing – review & editing. **Hui Xiao**: Investigation; Validation. **Chunxiuzi Liu**: Investigation; Visualization. **Shaohua Tang**: Investigation; Visualization. **Zhiwei Qin**: Funding acquisition; Writing – review & editing. **Jiakun Jiang**: Funding acquisition; Writing – review & editing. **Zengru Di**: Funding acquisition; Supervision; Writing – review & editing. **Yu Liu**: Conceptualization; Funding acquisition; Investigation; Methodology; Project administration; Resources; Supervision; Visualization; Writing – original draft; Writing – review & editing.

## CONFLICT OF INTEREST STATEMENT

The authors Lu Peng, Zecheng Zhang, Xianyi Wang, Weiyi Qiu, Liqian Zhou, Hui Xiao, Chunxiuzi Liu, Shaohua Tang, Zhiwei Qin, Jiakun Jiang, Zengru Di and Yu Liu declare that they have no conflict of interest or financial conflicts to disclose.

## ETHICS STATEMENT

This article is a review article and does not contain any studies with human or animal subjects performed by any of the authors.
